# Assessment of Machine Learning Model Performance for Clinical Prediction of Insulin Resistance in the Study of Cardiovascular Risk in Adolescents—ERICA

**DOI:** 10.3390/jcm15062224

**Published:** 2026-03-15

**Authors:** Jéssica Aparecida Silva, Katia Vergetti Bloch, Moyses Szklo, Rodolfo Deusdará

**Affiliations:** 1 Postgraduate Program in Medical Sciences, Faculty of Medicine, University of Brasília, Campus Universitário Darcy Ribeiro, Asa Norte, Brasília 70910-900, DF, Brazil; jeapsilva@poli.ufrj.br; 2 Institute for Studies in Public Health, Federal University of Rio de Janeiro, Cidade Universitária, Ilha do Fundão-Cidade Universitária, Rio de Janeiro 21941-598, RJ, Brazil; kbloch@iesc.ufrj.br; 3 Department of Epidemiology, Johns Hopkins Bloomberg School of Public Health, Baltimore, MD 21205, USA; mszklo1@jhu.edu; 4 Faculty of Medicine, University of Brasília, Campus Universitário Darcy Ribeiro, Asa Norte, Brasília 70910-900, DF, Brazil; 5 National Institute of Science and Technology for Health Technology Assessment (IATS), Porto Alegre 90035-903, RS, Brazil; 6 Laboratory of Epidemiology, Faculty of Medicine, University of Brasília, Campus Universitário Darcy Ribeiro, Asa Norte, Brasília 70910-900, DF, Brazil; 7 Institute for Health Assessment and Translation for Chronic and Neglected Diseases of High Relevance (IATS-CARE), Belo Horizonte 31270-901, MG, Brazil

**Keywords:** HOMA-IR, adolescents, machine learning, public health, insulin resistance

## Abstract

**Background**: Insulin resistance is defined as reduced tissue responsiveness to insulin-mediated glucose actions. Gold standard methods like hyperinsulinemic-euglycemic clamp and hyperglycemic clamps are costly and rarely used in large epidemiological studies. The aim was to evaluate the best performing machine learning algorithm for insulin resistance predictions in Brazilian adolescents. **Methods**: We used data from 37,454 Brazilian adolescents from 12 to 17 years, sampled from the Study of Cardiovascular Risk Factors in Adolescents (2013–2014). Covariates included other cardiovascular risk factors. We evaluate seven machine learning models stratifying the subset by sex. The performance of the models was assessed by area under the curve (AUC), calibration curves and decision curve analysis (DCA). Finally, we adopted the SHAP approach to assess the importance of each variable to the best performing ML model. **Results**: The Logistic Regression model presented the best AUC value (AUC = 0.8 for boys and girls). The best performing ML models had higher calibration in girls than in boys. The DCA curves showed prevalence of almost equal values for girls and for boys. The most important clinical predictors for both sexes were waist circumference, triglycerides and age. **Conclusions**: Logistic Regression proved to be the best clinical prediction model comparable to complex models. Further studies are needed in more diverse populations.

## 1. Introduction

Insulin resistance (IR) is defined as reduced tissue responsiveness to insulin-mediated glucose actions [1]. There are two gold standard methods for measuring IR, such as the hyperglycemic clamp and the hyperinsulinemic-euglycemic clamp. Nevertheless, these methods are costly, time-consuming, and therefore seldom applied in large-scale epidemiological studies [2]. In adolescents and adults, alternative indirect methods based on insulin and fasting glucose levels, including homeostasis model assessment of insulin resistance (HOMA-IR), are commonly employed [3,4,5]. In addition, there has been an increasing interest in the identification of cardiovascular disease (CVD) and type 2 diabetes mellitus (T2DM) in childhood and adolescence [6,7], and IR seems to be the common denominator between CVD and T2DM [8,9].

Recent studies have identified predictors associated with IR in adolescents and young adults, including obesity [1], low high-density lipoprotein (HDL) cholesterol [10,11], high low-density lipoprotein (LDL) cholesterol, high triglycerides [12], elevated waist circumference [13,14], and high blood pressure (BP) [15]. Additionally, unhealthy lifestyle factors such as sedentary behavior [16,17], alcohol consumption [18], and sugar-sweetened beverages [19] were associated with increased IR. It has been observed that smoking was positively associated with insulin resistance [20]. Regarding socioeconomic characteristics factors, although family income did directly affect HOMA-IR, parents’ education had a strong relation with IR in the adolescents in the study [21]. On the other hand, physical exercise improves insulin sensitivity [22,23]. A systematic review assessed the methodological performance of studies predicting the risk of undiagnosed type 2 diabetes mellitus (T2DM) or future risk. In this review, the most common predictors were age, family history of diabetes, body mass index, hypertension, waist circumference, and sex [24].

Artificial intelligence (AI) technologies have been effectively applied to disease diagnosis, personalized treatment, and prognosis [25,26]. In a Chinese study of children aged 6–12 years, five ML models were assessed to predict insulin resistance (IR), namely, Logistic Regression (LR), Support Vector Machine (SVM), Random Forest (RF), eXtreme Gradient Boosting (XGBoost), and Categorical Boosting (CatBoost) [27]. Among these ML models, XGBoost showed the largest area under curve (AUC, 0.85). Similarly, another Chinese study involving adults over 40 years old evaluated seven ML models, including LR, SVM, RF, ExtraTrees, Light Gradient-Boosting Machine (LightGBM), XGBoost, and Classification and Regression Tree (CART) [28]. LightGBM showed the highest predictive performance, with an AUC of 0.86. In a Korean study of adults over 40, seven ML models were evaluated, i.e., LR, XGBoost, Decision Tree (DT), K-Nearest Neighbors (KNN), SVM, RF, and Artificial Neural Network (ANN) [29]. LR and XGBoost performed best, each with an AUC of 0.86. A USA study with patients with chronic kidney disease over 18 years old evaluated four ML models: XGBoost, LR, Deep Neural Network (DNN), and RF [30]. XGBoost was the most effective, with an AUC of 0.78. Few studies that evaluated the predictive performance of ML models focused on populations from lower-middle-income countries, especially adolescents. Therefore, the present study aimed to develop the best predictive machine learning model for insulin resistance, defined by HOMA-IR, based on data from The Study of Cardiovascular Risk Factors in Adolescents (Portuguese acronym, ERICA) [31].

## 2. Materials and Methods

ERICA was a national school-based multi-center study conducted in 2013–2014 that aimed at providing estimates of cardiovascular risk factors, metabolic syndrome and its components in adolescents [31]. The study assessed the health conditions of around 75,000 students aged 12 and 17 years old from more than 1200 private and public schools, spread across 122 municipalities with more than 100,000 inhabitants. The 2011 School Census was used to select the sample of adolescents, and population was stratified into 32 geographical strata (27 capital cities and 5 macro-regions) [32].

### 2.1. Study Design

We selected a subsample of 37,815 adolescents in the morning shift, due to the requirement of a 12 h fasting period for collecting biochemical tests [31]. Data were stratified by gender, and all variables were obtained from adolescent questionnaires, parent questionnaires, school questionnaires, 24 h recall, anthropometric measurements, biochemical tests, and blood pressure [31]. The adolescent questionnaire was self-administered by the students using a Personal Digital Assistant (PDA), model LG GM750Q (LG Electronics, Seoul, Republic of Korea). Participants with missing values in the outcome or selected predictors were excluded, resulting in an analytic sample of 37,454.

### 2.2. Outcome

The outcome was HOMA-IR, calculated as insulin (mU/L) × (glucose (mg/dL) × 0.0555)/22.5 [4]. Chissini et al. [33] analyzed ERICA and showed that the ideal cut-off for HOMA-IR associated with metabolic syndrome was 2.8 for the total population, 2.32 for boys, and 2.87 for girls. A binary variable for insulin resistance was created based on these cutting points.

### 2.3. Predictors

#### 2.3.1. Biochemical Assays

Biochemical analyses were performed at the study reference laboratory in Cascavel, PR, Brazil, which followed the required quality control standards. A detailed description of blood collection quality control procedures has been published elsewhere [19]. HDL cholesterol and triglyceride levels were measured using the enzymatic colorimetric and enzymatic kinetic assays, respectively. HDL cholesterol was measured in the plasma level of HDL (mg/dL) in the fasting state. Low HDL was defined as <45 mg/dL for boys and girls [31]. Triglyceride was measured in the fasting plasma level of triglycerides (mg/dL). High triglyceride was defined as equal to or greater than 150 mg/dL [31]. LDL cholesterol was calculated using the Friedewald equation [cholesterol_hdl + (triglycerides/5)], only for those with cholesterol of less than 400 mg/dL [34].

#### 2.3.2. Anthropometric Measures

Adolescents wore no shoes and light clothes during the anthropometric measurements. Height was measured twice using Alturexata^®^ stadiometer (Alturexata, Ouro Preto, MG, Brazil) with 1 mm resolution and a maximum height of 213 cm. A maximum variation of 0.5 cm was allowed; if the difference exceeded this limit, the measurements were repeated. Weight was measured using a Líder^®^ digital scale (Líder Balanças, Araçatuba, SP, Brazil), model P150m, with a capacity of 200 kg and 50 g resolution [31].

Waist circumference was measured twice at the midpoint between the lower curve of the last fixed rib and the upper curve of the iliac crest with the adolescent standing, arms beside the body, feet together, and abdomen relaxed, using a Sanny^®^ fiberglass anthropometric tape (Sanny, São Paulo, SP, Brazil) [31].

Nutritional status was based on the body mass index (BMI), defined as weight in kilograms divided by the square of height in meters. The classification of nutritional status was determined by the World Health Organization, which considers BMI by age and sex for adolescents using Z-scores. Obesity, overweight, normal, underweight and low weight nutritional status were defined as follows: Z-score > +2, Z-score > +1 and Z-score ≤ +2, Z-score ≥ −2 and Z-score ≤ +1, Z-score < −3, and Z-score ≥ −3 and Z-score < −2, respectively [35].

#### 2.3.3. Health Lifestyle

Physical inactivity was based on a checklist of 24 questions related to the week before the day of the interview. The adolescent was considered inactive if the time spent in the activity was less than or equal to 420 min/week [31].

Smoking habit was defined as having smoked at least one cigarette in the past 30 days.

Alcohol consumption was defined as at least one dose of alcohol in the last 30 days.

Sedentary behavior was defined as more than 3 h a day spent with computer, television, and video game use on an average weekday.

Blood pressure (BP) was measured twice using an Omron^®^ 705-CP device (Omron Healthcare Co., Ltd., Kyoto, Japan) and was based on the Fourth Report on the Diagnosis, Evaluation, and Treatment of High Blood Pressure in Children and Adolescents [28]. Hypertension was defined as 95th percentile for gender, age, and height [31].

Socioeconomic status was defined by whether the adolescent attended public or private school.

### 2.4. Statistical Analysis

For descriptive statistics of the dataset, absolute (raw counts) and relative frequencies were presented for categorical variables, and measures of central tendency (mean) and variation (standard deviation) were presented for continuous variables with a normal distribution. Medians and interquartile ranges were presented for continuous variables with non-normal distribution.

The sample with 37,454 adolescents was stratified by sex, where each group was randomly split to form the training and testing datasets for the algorithms. To ensure generalizability and to avoid overfitting, each group (girls and boys) was divided to maintain the same proportion of the outcome variable in the training and test subsets.

Model performance was evaluated using the area under the receiver operating characteristic curve (AUC) and other metrics, such as sensitivity, specificity, F1-score, positive predictive value (PPV), and negative predictive value (NPV). Sensitivity and specificity were computed using a fixed probability threshold of 0.50 for classification. The algorithms were evaluated on a test subset containing samples that were not used in the training dataset [36].

To assess the contribution of each variable to the best-performing model, we used the SHapley Additive exPlanations (SHAP) approach, which provides insight into the predictive importance of each variable and the direction (positive or negative) of individual variable values [37]. We analyzed the calibration curve of the probabilities predicted by each model and the probability predictions, which should be well calibrated and close to the perfect calibration curve [26]. Finally, we analyzed the model’s performance compared to conventional treatment strategies using decision curve analysis (DCA), considering the use of the HOMA-IR clinical indicator to detect insulin resistance [38].

We conducted a sensitivity analysis comparing complex and non-complex survey designs in the development of the Logistic Regression model. We used a HOMA-IR cut-off value of 2.63 for girls and 2.28 for boys.

All analyses were conducted using R version 4.1.2 (R Core Team, Vienna, Austria). The aim was to utilize open-source tools to minimize implementation costs for technological projects [39]. The local ethics committees approved the study, and informed consent was obtained from all individuals and their parents or legal guardians included in this study.

### 2.5. Preprocessing, Data Splitting and Model Building

Classification models were developed to predict insulin resistance (IR), a binary variable. We divided the sample of 37,454 by sex, and each subgroup was partitioned into a training dataset (70% subsample) and a testing dataset (30% subsample). The training dataset was used to define the model’s parameters (input variables) and hyperparameters (settings outside the model), while the test dataset was used to evaluate the model performance using data that had not been seen before.

Prior to model training, preprocessing procedures were applied. Numerical variables were normalized using the Min–Max scaling method, which rescales values to a range between 0 and 1 to ensure comparable scales across predictors. Categorical variables were transformed into numerical representations to allow their inclusion in the machine learning models. Subsequently, one-hot encoding was applied, creating binary indicator variables for each category.

Several predictors were defined using clinically established cut-off points based on prior literature, as described in Section 2.2 and Section 2.3. These categorized variables included nutritional status, sedentary behavior, smoking status, alcohol consumption, blood pressure classification, and insulin resistance. In our dataset, the proportion of missing data was very low across all predictors. The highest level of missingness was observed for HOMA-IR (0.76%), followed by LDL cholesterol (0.32%), HDL cholesterol (0.28%), triglycerides (0.29%), and waist circumference (0.15%). Therefore, no variables exceeded the original 1% exclusion threshold, and no predictors were removed from the analysis due to missing data.

**Figure 1:**
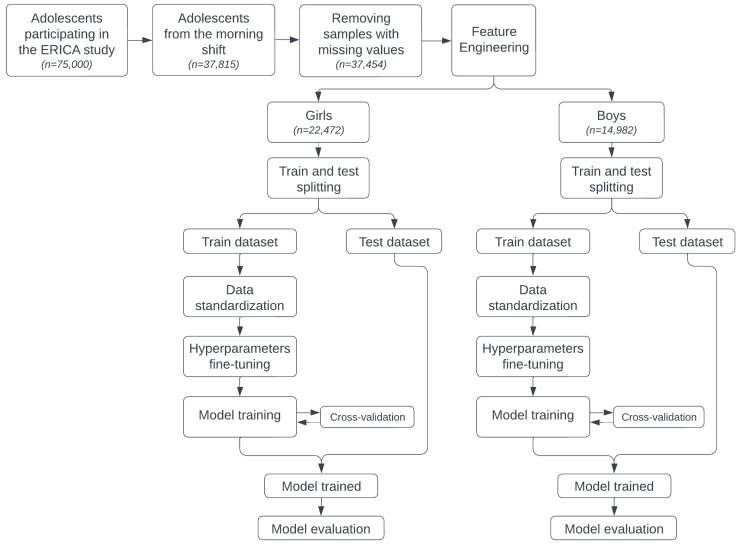
Workflow for training machine learning models to predict insulin resistance in the Study of Cardiovascular Risk Factors in Adolescents (ERICA, 2013–2014). Out of 37,815 potentially eligible participants for model development, 37,454 adolescents met the inclusion criteria, consisting of students from the morning shift.

In addition, we used repeated random subsampling validation with ten independent iterations to train each model. The most common binary classification algorithms were tested, including the traditional Logistic Regression (LR) and Poisson, and the AI approaches Decision Tree (DT), Random Forest (RF), Support Vector Machine (SVM), eXtreme Gradient Boosting (XGBoost), and Deep Neural Network (DNN) [26]. Figure 1 shows all the steps involved in building the machine learning models.

## 3. Results

### 3.1. Description of the Study Population

We identified 37,454 adolescents potentially eligible for ML models, which were morning shift adolescents due to the requirement of a 12 h fasting period for collection and adolescents who had all input variables (Figure 1). The median age of our study sample was 15 years, ranging from 12 to 17 years. Almost 20% of all adolescents were classified as having insulin resistance and a similar proportion had excess weight (overweight plus obesity), with obesity accounting for close to 9%. About 20% of adolescents had consumed at least one alcoholic drink in the previous 30 days, and less than 4% had smoked at least one cigarette in the previous 30 days. Approximately 40% of adolescents had sedentary behavior. Most adolescents were physically inactive, enrolled in public schools, and had a normal waist circumference (WC). Of the lipid markers, HDL cholesterol showed the least inter-student variability (Table 1).

### 3.2. Evaluation of the Models’ Performance

#### 3.2.1. Comparison of AUC Curve and Model’s Metrics

**Figure 2:**
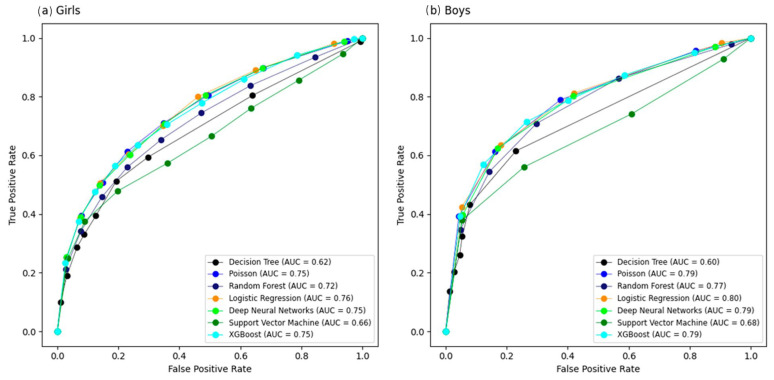
Area under the curve (AUC) of machine learning algorithms for predicting insulin resistance in ERICA (2013–2014), calculated using the test dataset: (**a**) girls; (**b**) boys. Abbreviations: XGBoost, eXtreme Gradient Boosting; AUC, area under the receiver operating characteristic curve.

We evaluated the performance of seven machine learning models stratified by sex. Analysis of the AUC curve for the girls showed that the AUC for five models (LR, Poisson, XGBoost, DNN, and RF) ranged between 0.72 and 0.76, while for the boys, the same models produced values ranging from 0.77 to 0.80 (Figure 2). These five models showed false positive proportions that ranged between 0.26 and 0.34 for girls and 0.26–0.36 for boys. Sensitivity levels were between 0.19 and 0.22 for girls, and between 0.14 and 0.31 for boys. Table 2 shows the best results for each algorithm in terms of AUC, sensitivity, specificity, positive predictive value (PPV), negative predictive value (NPV), and F1-score metrics. In a sensitivity analysis, we tested the complex sampling design and alternative sex-specific HOMA-IR cut-off values. These analyses did not change our conclusions (Appendix A Table A1).

#### 3.2.2. Comparison of Calibration Curve

**Figure 3:**
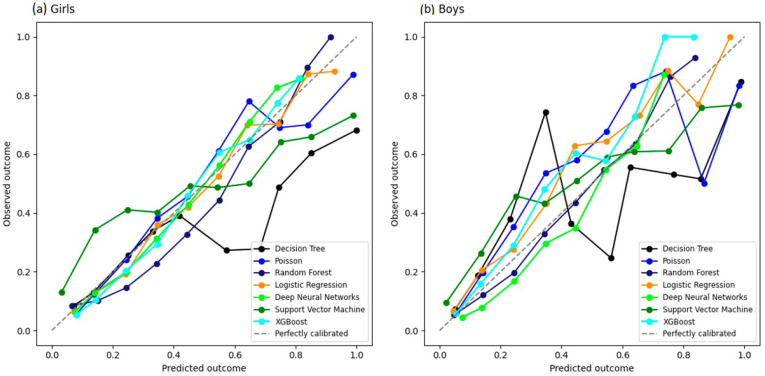
Calibration curve analysis of machine learning algorithms for predicting insulin resistance in ERICA (2013–2014), calculated using the test dataset: (**a**) girls; (**b**) boys.

We plotted calibration curves for all models (Figure 3) using the test dataset. Visual inspection of the calibration curves suggested that LR, XGBoost, and DNN were closer to the 45° line in girls than in boys.

#### 3.2.3. Comparison of Decision Curve Analysis

**Figure 4:**
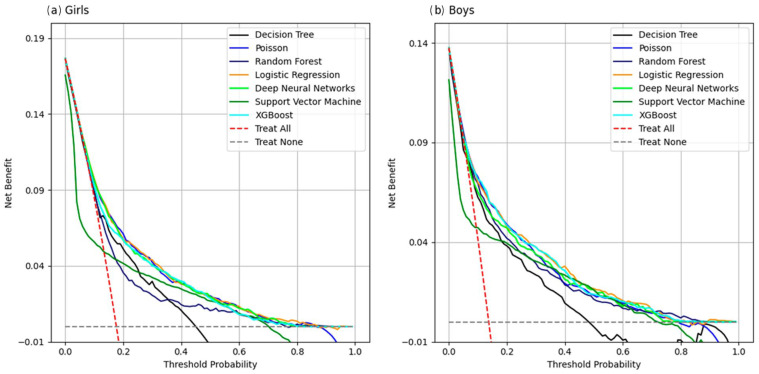
Decision curve analysis (DCA) of machine learning algorithms for predicting insulin resistance in ERICA (2013–2014), calculated using the test dataset: (**a**) girls; (**b**) boys.

In girls, the DCA analysis showed that for threshold probabilities of around 18%, the SVM model had a lower net benefit than the option of seeking clinical intervention. On the other hand, for values above this threshold, the DT and RF models showed the lowest benefit of clinical intervention compared to the other models. In boys, the threshold probability was 16%, lower than that found in the girls, and the SVM model showed the same behavior of not proving to be as beneficial as clinical intervention. Beyond this threshold, only the DT model showed the lowest net benefit compared to the others (Figure 4).

#### 3.2.4. Analysis of the Importance of Clinical Predictors

**Figure 5:**
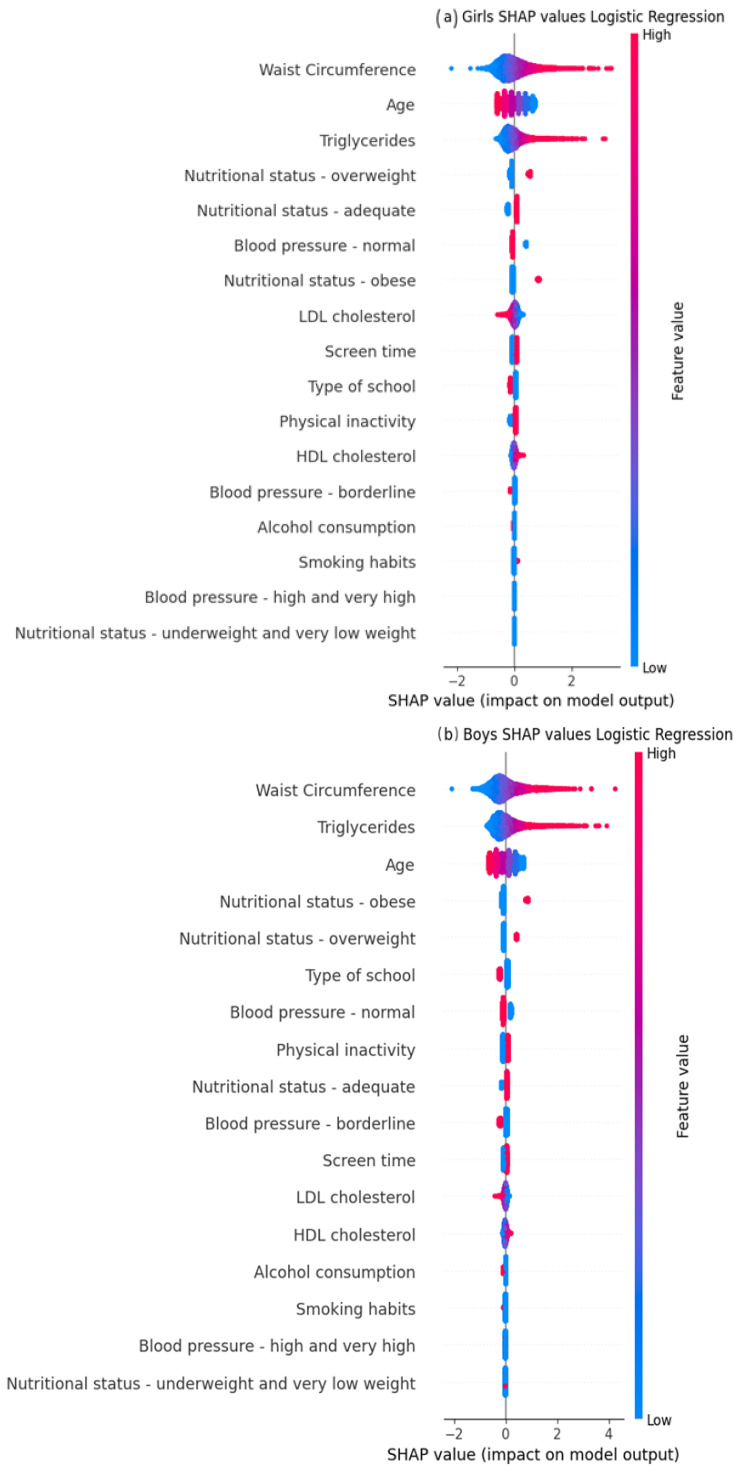
Variable importance from Shapley values for the Logistic Regression model in the ERICA (2013–2014) test dataset: (**a**) girls; (**b**) boys. Red color indicates higher contributions, blue lower contributions.

We evaluated the clinical predictors importance of the LR model in boys and girls. For both boys and girls, the top three ranked clinical predictors were waist circumference, triglycerides, and age (Figure 5).

## 4. Discussion

This study was the first to evaluate and develop machine learning to predict insulin resistance in Brazilian adolescents. In our study, AUC analysis showed that five models (LR, Poisson, XGBoost, DNN, and RF) had good performance in adolescents from ERICA, ranging from 0.72 in RF for girls to 0.80 in LR for both boys and girls. These differences in the AUC metric may have been due to hormonal differences between boys and girls during adolescence. Girls tended to enter puberty earlier, which increased estrogen levels and caused fat to accumulate in areas such as the hips and thighs, while in boys, testosterone promoted the accumulation of abdominal fat. In addition, growth patterns differ between boys and girls and could have affected waist circumference measurements at different ages and stages of development [40,41]. Looking at the Shapley Graphic (Figure 5), it was observed that the effect of waist circumference was greater in the LR model for boys, indicating that the estimates may have been affected by these differences.

Moreover, the five models (LR, Poisson, XGBoost, DNN, and RF) exhibited a considerable false positive proportion (0.26–0.36) and low sensitivity (0.14–0.31). These findings suggest that while the models are effective at identifying adolescents without IR, they showed limited ability to detect adolescents with IR when using the same classification rule across models. Sensitivity and specificity are threshold-dependent; in this study, these metrics were calculated using a fixed probability threshold of 0.50 to ensure comparability across models. The prevalence of insulin resistance in our sample was 19.8% (Q1 = 19.4%, Q3 = 20.2%), and this class distribution may contribute to lower sensitivity when standard thresholds are applied. For screening purposes, alternative thresholds can be selected to prioritize sensitivity, with an expected trade-off in specificity. In addition, three machine learning models (LR, XGBoost and DNN) have higher calibration in girls than in boys. Finally, the decision curve analysis (DCA) suggested potential clinical utility for decision thresholds above the observed prevalence for most models, except SVM. One possible explanation is that the cumulative effects of risk factors related to insulin resistance may become evident only after several years of exposure, which may partially reduce detectability in adolescents.

Our study is consistent with literature studies that evaluate the performance of clinical prediction of insulin resistance based on machine learning models because the AUC values ranged from 0.75 to 0.85 [27,28,29,30]. In a Chinese study with 503 children aged 6 to 12 years old, the XGBoost model achieved a performance of AUC = 0.85, with age, hip circumference and waist circumference as the main variables. In addition, Lee et al. [30] developed models for the population of adults over 18 years with chronic kidney disease and found that the XGBoost model achieved AUC = 0.78, with BMI, age and triglycerides as the main variables. Finally, Hao Zhang et al. [28] developed models for adults over 40 years old. The best-performing model was LightGBM, with AUC = 0.79, and the main clinical variables for prediction were waist circumference, BMI and fasting plasma glucose.

To select the optimal machine learning model for predicting insulin resistance, choosing a parsimonious model that balances model complexity with predictive ability is necessary. Logistic regression is the most suitable choice in this context due to its interpretability, broad applicability in clinical settings, computational efficiency, and comparable performance to other developed models [42]. In our study, waist circumference, triglyceride levels, and age were key predictors for clinical prediction in girls and boys when using the logistic regression model.

Looking at the ranking of the three main variables in the models, our study also included variables that have already been included in other studies, such as waist circumference [27,28,29], triglycerides [28,30] and age [27,30], which contribute most to the prediction of insulin resistance.

From a clinical perspective, this model can be used as a decision-support tool for risk stratification, and not as a diagnostic replacement. It could be implemented in primary care or school-based screening settings to identify adolescents at higher risk of insulin resistance and prioritize confirmatory testing and follow-up. Since the predictors are based on routinely collected measurements and questionnaire data, the approach has potential for scalability and low implementation costs. In addition, the probability threshold can be adjusted depending on the intended use (e.g., screening to prioritize sensitivity). Finally, external validation in independent cohorts is needed before clinical implementation.

Our study was cross-sectional and therefore subject to selection/survival and temporal bias. However, survival bias is unlikely, given the young age of our study sample. On the other hand, despite the biological plausibility of our results, temporal bias may have occurred. In addition, social desirability bias may have occurred in the collection of questionnaires from adolescents, parents, and school. Another potential limitation is that information bias may have occurred, because some predictors were identified mostly by self-reported behavioral data, which may introduce measurement bias. Residual confounding may have occurred despite multivariable adjustment for all single observational studies. In addition, using a binary outcome variable may result in a loss of information. However, dichotomization is helpful in clinical practice because it allows the distinction between “abnormal” from “normal” results (the cost of dichotomizing continuous variables [40]).

Our study did not use a complex survey design to build the machine learning models because the R programming language do not allow this implementation. However, sensitivity analysis with and without complex survey design in the LR regression model did not change our results (Appendix A Table A1). Future studies should address the incorporation of complex survey designs into machine learning models and provide new tools for prediction using machine learning models.

## 5. Conclusions

Our study demonstrates that the LR model could be useful for predicting insulin resistance in ERICA adolescents. When we analyzed the AUC of the models, we found that LR, Poisson, XGBoost, DNN, and RF performed best for both boys and girls. In the calibration curve of the predicted probabilities, the LR, XGBoost and DNN models also stood out for both sexes. In addition, LR, DNN, XGBoost and Poisson were the only models able to contribute to clinical intervention above the prevalence threshold, regardless of gender. We chose a parsimonious model to select the optimal machine learning model for predicting insulin resistance. Due to its interpretability, broad clinical applicability, computational efficiency, and similar performance to other models, LR represents the most appropriate choice in this context. Future studies should develop methodologies that account for complex sampling designs for clinical prediction using machine learning models.

## Figures and Tables

**Table 1:** Characteristics of 37,454 Brazilian participants enrolled in the Study of Cardiovascular Risk Factors in Adolescents (ERICA, 2013–2014).

**Variables**	***n***			
**Continuous**		**Median**	**1º*Q***	**3º*Q***
Age (years)	37,454	15	13	16
Waist Circumference (cm)	37,454	69.9	65.1	76.1
HDL cholesterol	37,454	45.8	39.5	53.3
LDL cholesterol	37,454	83.6	70.1	99.1
Triglycerides	37,454	70	54	92
**Categorical**		**(%)**	**95% CI**	
Girls	22,682	60	59.5	60.5
Sedentary behavior ^Φ^	14,133	52.3	51.8	52.8
Type of school	37,454			
Public		74	73.6	74.5
Private		26	25.5	26.4
Nutritional status	37,454			
Normal ^a^		71.7	71.2	72.1
Overweight ^b^		17.5	17.2	17.9
Obesity ^c^		8.1	78.7	8.4
Underweight and low weight ^d^		2.7	2.5	2.8
Blood Pressure	37,454			
Normal		77.8	77.4	78.2
Borderline		13.1	12.8	13.5
High and very high		9	8.8	9.4
Smoking ^Ω^	1406	3.7	3.5	3.9
Alcohol consumption (≥1 drink in the last 30 days)	7685	20.4	20	20.8
Physical inactivity (<420 in the last week)	24,713	65.3	64.8	65.8
Insulin resistance ^µ^	7423	19.8	19.4	20.2

^Φ^ ≥3 h a day spent with television, video games, or a computer on an ordinary weekday; ^a^ Z-score ≥ −2 and Z-score ≤ +1; ^b^ Z-score > +1 and Z-score ≤ +2; ^c^ Z-score > +2; ^d^ Z-score < −3, Z-score ≥ −3 and Z-score < −2; ^Ω^ smoked at least one cigarette in the past 30 days; ^µ^ IR was defined based on HOMA-IR, with a cut-off of 2.32 for boys and 2.87 for girls. Abbreviations: *n*, size sample; 1º*Q*, first quartile; 3º*Q*, third quartile.

**Table 2:** Performance metrics of machine learning models for predicting insulin resistance according to sex in ERICA (2013–2014).

**Girls**						
**Model**	**AUC (95% CI)**	**Sensitivity**	**Specificity**	**F1–Score**	**PPV**	**NPV**
Logistic Regression	0.80 (0.77–0.82)	0.19	0.99	0.30	0.74	0.88
Poisson	0.75 (0.74–0.77)	0.19	0.98	0.31	0.71	0.85
Deep Neural Network	0.75 (0.73–0.77)	0.23	0.97	0.34	0.66	0.86
XGBoost	0.75 (0.73–0.76)	0.22	0.97	0.33	0.66	0.85
Random Forest	0.72 (0.70–0.73)	0.26	0.96	0.36	0.56	0.86
SVM	0.66 (0.64–0.68)	0.21	0.98	0.31	0.65	0.85
Decision Tree	0.62 (0.60–0.63)	0.32	0.92	0.37	0.45	0.86
**Boys**						
**Model**	**AUC (95% CI)**	**Sensitivity**	**Specificity**	**F1–Score**	**PPV**	**NPV**
Logistic Regression	0.80 (0.77–0.82)	0.19	0.99	0.30	0.74	0.88
Deep Neural Network	0.79 (0.77–0.81)	0.31	0.97	0.41	0.62	0.9
XGBoost	0.79 (0.77–0.81)	0.16	0.99	0.26	0.73	0.88
Poisson	0.79 (0.77–0.81)	0.14	0.99	0.23	0.78	0.88
Random Forest	0.77 (0.75–0.79)	0.19	0.98	0.29	0.64	0.88
SVM	0.68 (0.66–0.71)	0.23	0.98	0.34	0.67	0.89
Decision Tree	0.60 (0.59–0.62)	0.25	0.96	0.33	0.48	0.89

Abbreviations: XGBoost, eXtreme Gradient Boosting; SVM, Support Vector Machine; AUC, area under the receiver operating characteristic curve; PPV, positive predictive value; NPV, negative predictive value.

## Data Availability

The data are available upon request.

## Abbreviations

The following abbreviations are used in this manuscript: AUC, area under the receiver operating characteristic curve; AI, artificial intelligence; ANN, Artificial Neural Network; BMI, body mass index; BP, blood pressure; CVD, cardiovascular disease; DCA, decision curve analysis; DNN, Deep Neural Network; DT, Decision Tree; ERICA, Study of Cardiovascular Risk Factors in Adolescents; HDL cholesterol, high-density lipoprotein cholesterol; HOMA-IR, homeostasis model assessment of insulin resistance; IR, insulin resistance; IQR, interquartile range; LDL cholesterol, low-density lipoprotein cholesterol; LR, Logistic Regression; ML, machine learning; NPV, negative predictive value; PPV, positive predictive value; RF, Random Forest; SHAP, SHapley Additive exPlanations; SVM, Support Vector Machine; T2DM, type 2 diabetes mellitus; XGBoost, eXtreme Gradient Boosting.

## Appendix A

**Table A1:** Comparison of logistic regression models between simple and complex survey designs and variation in the cut-off value for girls (2.63) and boys (2.28).

**Girls**						
**Model**	**AUC (95% CI)**	**Sensitivity**	**Specificity**	**F1-Score**	**PPV**	**NPV**
Logistic Regression Simple Survey Design	0.80	0.19	0.99	0.30	0.74	0.88
	(0.77–0.82)					
Logistic Regression Complex Survey Design	0.75	0.23	0.97	0.33	0.64	0.85
	(0.73–0.77)					
Logistic Regression with cut-off of 2.63 for girls and 2.28 for boys	0.74	0.23	0.97	0.34	0.65	0.81
	(0.73–0.76)					
**Boys**						
**Model**	**AUC (95% CI)**	**Sensitivity**	**Specificity**	**F1-score**	**PPV**	**NPV**
Logistic Regression Simple Survey Design	0.80	0.19	0.99	0.30	0.74	0.88
	(0.77–0.82)					
Logistic Regression with cut-off of 2.63 for girls and 2.28 for boys	0.75	0.39	0.94	0.49	0.67	0.83
	(0.74–0.77)					
Logistic Regression Complex Survey Design	0.75	0.21	0.99	0.32	0.74	0.89
	(0.73–0.77)					
